# Whole genome sequencing of the fish pathogen Francisella noatunensis subsp. orientalis Toba04 gives novel insights into Francisella evolution and pathogenecity

**DOI:** 10.1186/1471-2164-13-598

**Published:** 2012-11-06

**Authors:** Settu Sridhar, Animesh Sharma, Heidi Kongshaug, Frank Nilsen, Inge Jonassen

**Affiliations:** 1 Department of Informatics, University of Bergen, Bergen, Norway; 2 Department of Biology, University of Bergen, Bergen, Norway; 3 Computational Biology Unit, Uni computing, University of Bergen, Bergen, Norway

**Keywords:** *Francisella*, Macrophage, Pseudogenes, Genome, Insertion elements, Comparative analysis

## Abstract

**Background:**

*Francisella* is a genus of gram-negative bacterium highly virulent in fishes and human where *F. tularensis* is causing the serious disease tularaemia in human. Recently *Francisella* species have been reported to cause mortality in aquaculture species like Atlantic cod and tilapia. We have completed the sequencing and draft assembly of the *Francisella noatunensis subsp. orientalisToba04 strain* isolated from farmed Tilapia. Compared to other available *Francisella* genomes, it is most similar to the genome of *Francisella philomiragia subsp. philomiragia*, a free-living bacterium not virulent to human.

**Results:**

The genome is rearranged compared to the available *Francisella* genomes even though we found no IS-elements in the genome. Nearly 16% percent of the predicted ORFs are pseudogenes. Computational pathway analysis indicates that a number of the metabolic pathways are disrupted due to pseudogenes. Comparing the novel genome with other available *Francisella* genomes, we found around 2.5% of unique genes present in *Francisella noatunensis subsp. orientalis Toba04* and a list of genes uniquely present in the human-pathogenic *Francisella subspecies*. Most of these genes might have transferred from bacterial species through horizontal gene transfer. Comparative analysis between human and fish pathogen also provide insights into genes responsible for pathogenecity. Our analysis of pseudogenes indicates that the evolution of *Francisella* subspecies’s pseudogenes from Tilapia is old with large number of pseudogenes having more than one inactivating mutation.

**Conclusions:**

The fish pathogen has lost non-essential genes some time ago. Evolutionary analysis of the *Francisella* genomes, strongly suggests that human and fish pathogenic *Francisella* species have evolved independently from free-living metabolically competent *Francisella* species. These findings will contribute to understanding the evolution of *Francisella* species and pathogenesis.

## Background

Species in the *Francisella* genus are facultative intracellular, gram-negative bacteria, and well known for causing Tularaemia in mammals. *Francisella* was first found by the American bacteriologist Edward Francis in 1922 [1]. The *Francisella tularensis* subspecies strains can be serious pathogens for human and can cause tularaemia that lead to mortality, making these bacteria a potential bio-weapon [2]. Until recently the *Francisella* genus only consisted of two species, *F. tularensis subsp*. *tularensis* and *F. philomiragia subsp. philomiragia* where *Francisella philomiragia subsp. Philomiragia* is a non-virulent species. Recently, more *Francisella* species and strains have been isolated from several new sources. From farmed Atlantic cod, a new and highly virulent species of *Francisella* was recently described and has later been given the name *F.noatunensis subsp. noatunensis*[3,4]. *Francisella* has also been reported from other fish species and another fish pathogenic strain, *F. noatunensis subsp. orientalis Toba04* has been obtained from tilapia [4-6]. In addition, *Francisella* has been identified in environmental samples and from invertebrates like ticks [2,7]. Although the available *Francisella* genomes are fairly close to each other in their features, their genomes are highly rearranged [8]. The molecular phylogeny of *Francisella* species and strains has been reported previously and the family *Francisellaceae* currently contains one genus only and there is no close pathogenic relative to this bacteria family [9]. The subspecies of *F. tularensis* are classified into human virulent, non-virulent, and moderately virulent. *F. tularensis subsp. tularensis* and *F. tularensis subsp. holarctica* are human virulent strains, the latter being less virulent [9,10]. *F. tularensis subsp. mediasiatica* is moderately virulent to human [11]. *F. tularensis subsp. novicida and F. philomiragia subsp. philomiragia* are not virulent to human. The *F. noatunensis* strains are not able to grow at 37DC and hence they are not virulent to human [5].

The *Francisella* species can also be categorized into metabolically competent and metabolically incompetent. The metabolically competent strains have been found in environmental samples while the incompetent depend on a host for growth. The metabolic competence of a species relate to the number of intact metabolic genes present in its genome [10]. *F. tularensis subsp. tularensis*, *F. tularensis subsp. holarctica* and *F. tularensis subsp. mediasiatica* are metabolically incompetent and have a larger number of disrupted genes (i.e. partially conserved genes with internal stop codons or frameshifts) in their genomes while *F. philomiragia subsp. philomiragia* and *F. tularensis subsp. novicida* are metabolically competent and have few disrupted genes. The characterized genomes of strains within the *F. tularensis* subspecies are highly rearranged between themselves and insertion elements (IS-elements) have been regarded as a key feature to create these rearrangements. The genomic breakpoints are typically flanked by IS-elements and associated with a large number of pseudogenes [8-10]. The *F. tularensis* subspecies genomes possesses two copies of FPI (Francisella pathogenecity island), while the metabolically competent have one copy of FPI. Although several studies comparing human virulent, moderately virulent strains and non-virulent strains have been reported, the mechanisms behind the pathogenecity of *Francisella* strains are still largely unknown.

Identification of several new highly virulent strains of *Francisella* from farmed fish has opened up for a broader comparison between members of this important and very special group of bacteria. The *F. noatunensis* strains are highly pathogenic to fishes and can cause high mortality and losses in farmed fish [12]. To gain more detailed information about *F. noatunensis* subspecies we sequenced *F. noatunensis subsp. orientalis Toba04 strain* genome using pyrosequencing. We were able to assemble the *F. noatunensis subsp. orientalis Toba04* genome into one high quality scaffold. The genome sequence and assembly of the virulent fish pathogen *F. noatunensis subsp. orientalisToba04* has been annotated and used in a comparative genomic approach to analyze the properties that are shared with the mammalian pathogenic and environmental *Francisella* strains. We also tried to understand the factor influencing virulence among the *Francisella* strains used for comparative studies. Our sequence analysis revealed that the *F. noatunensis subsp*. *orientalis Toba04* strain lack IS-elements shedding new light on the role and possibly the mechanisms of genome rearrangement in the *Francisella* species. One feature shared between the *orientalis* and the human pathogenic *Francisella* strains is the presence of disrupted genes and metabolic incompetence.

## Results and discussion

### Genome features

We were able to assemble the genomic reads for *F. noatunensis subsp*. *orientalis Toba04* (Genbank accession number NC_017909) into one contig with a size of 1.84 Mbp (this included some targeted sequencing to perform gap closure; see Methods for details). We have predicted 1595 protein coding genes in the *F. noatunensis subsp. orientalis Toba04* genome and most of the encoded (putative) proteins are most similar to proteins encoded in the *F. philomiragia subsp. philomiragia* genome (80-100% identical on amino acid level) followed by *F. tularensis subsp. novicida*. The fish pathogenic *Francisella* genome share properties both with virulent and non-virulent *Francisella* species (Table 1). The parasitic *Francisella* species have in general a more compact genome with fewer protein coding genes and with a relatively large number of pseudogenes compared to *F. philomiragia subsp*. *philomiragia* and *F. tularensis subsp*. *novicida* (Additional file 1: Figure S1). A striking difference between the fish parasitic *Francisella* and the mammalian parasites is the lack of insertion elements (IS elements) and only one copy of the pathogenecity island (FPI) in the fish parasite.

**Table 1:** **Summarizing some main characteristics for the genome sequence presented in this paper together with a representative set of other already sequenced *Francisella *genomes**

	***F. noatunensis subsp. Orientalis Toba04***	***F. philomiragia subsp. philomiragia ATCC 25017***	***F. tularensis subsp. novicida U112***	***F. tularensis subsp. medisisatica FSC 147***	***F.tularensis subsp. tularensis SCHU S4***	***F.tularensis subsp. holarctica OSU18***
	**Fish parasite**	**Free-living**	**Free-living**	**Mammalian parasite**	**Mammalian parasite**	**Mammalian parasite**
**Genome size(bp)**	**1,847,202**	**2,045,775**	**1,910.031**	**1,893,886**	**1,892,775**	**1,895,727**
**GC content(%)**	**32**	**32**	**32**	**32**	**32**	**32**
**ORFs**	**2289**	**1966**	**1781**	**1750**	**1852**	**1932**
**Protein coding genes**	**1595**	**1911**	**1719**	**1406**	**1604**	**1555**
**Structural RNAs**	**39**	**48**	**48**	**47**	**48**	**49**
**IS elements**	**0**	**8**	**29**	**85**	**78**	**116**
**Pathogenecity Island**	**1**	**1**	**1**	**2**	**2**	**2**
**Pseudogenes**	**252**	**3**	**14**	**297**	**200**	**328**

### Francisella became pathogenic long before it became pathogenic to mammals

**Figure 1:**
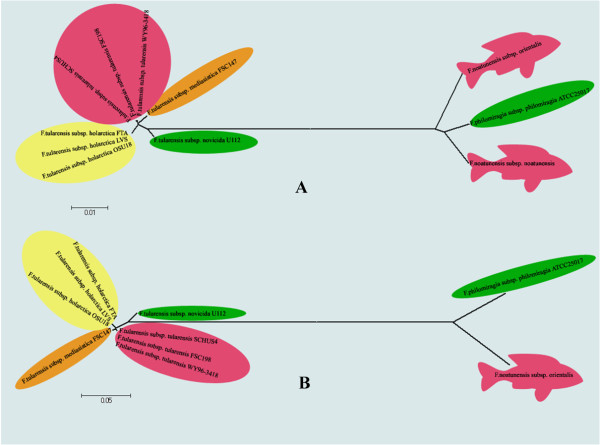
**A: Phylogenetic tree made using core genes present in *Francisella noatunensis subsp. orientalis Toba04 *and *Francisella noatunensis subsp. noatunensis *(from atlantic cod; incomplete assembly). B:** Whole genome phylogenetic tree made from available *Francisella* subspecies. Red colour represents highly virulent species while yellow represent less virulent ones. Orange represents moderately virulent species and green represent very rarely or non-virulent ones.

Several studies have addressed the molecular phylogeny of *Francisella* species and subspecies [6,10] and we conducted a whole genome phylogenetic analysis based on all available *Francisella* genomes [Figure 1B and a tree based on the core set of genes present in the *Francisella* species [Figure 1A. We were able to find all the core genes from *F. noatunensis subsp. noatunensis* in the incomplete assembly from our sequence data and included that strain only to build the tree. This analysis confirms previous studies that there are two main groups of *Francisella*, the *F. tularensis subspecies* group and the *F. philomiragia* subspecies group, where the fish parasitic species belong to the latter. The phylogenetic distance between the species in the “*Philomiragia* group” is larger than between the species in the *Tularensis* group. However, the distance between the two groups is quite large. Hence, neither this nor other studies show a close phylogenetic link between the different pathogenic species within the *Francisella* genus. It appears likely that the mammalian pathogenic species and the fish pathogenic species result from (at least two) independent events of a free-living *Francisella* ancestral species acquiring a pathogenic lifestyle, and the phylogeny analysis is consistent with the fish pathogenic *Francisella* being result of an ancient event resulting in the genome containing no IS elements. This result is also supported by the recent publication conducting evolutionary analysis between fish and mammalian pathogens [4].

The lack of IS elements in the genome of *F. noatunensis subsp*. *orientalis Toba04* suggests that its acquisition of a pathogenic lifestyle happened a long time ago. Moran and Plague found that pathogens or symbionts that have recently adopted an obligate host association have numerous IS elements while ancient obligate host associations most often have no IS elements [13]. This suggests that the present *Francisella* strain pathogenic to tilapia results from an ancient event where free-living *Francisella*-like bacteria infected fish (possibly tilapia) and underwent a period of gene decay and genome rearrangement where IS elements may have played a major role. As observed by Moran and Plague, bacteria that have ancient obligate host associations, lack IS-elements, and the present data on *F. noatunensis subsp. orientalis Toba04* suggests that it falls into this category. This is also supported by the results of our analyses of phylogeny and pseudogenes content summarized below.

The two fish infecting *Francisella* does not form a monophyletic group (Figure 1A) and this is an indication that they can have become parasitic at two independent events. This further indicates that they have lost there IS-elements independently and this view is supported by the fact that the two fish parasitic bacteria have different re-arrangements in their genomes.

### Francisella species have highly rearranged genomes

**Figure 2:**
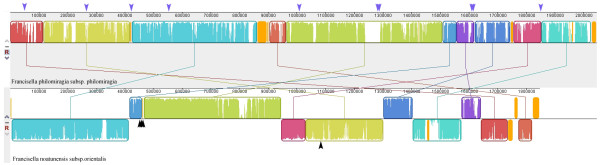
**The following figure shows the rearrangement plot between *Francisella philomiragia subsp. philomiragia ATCC25017 *and *Francisella noatunensis subsp. orientalis Toba04*.** The purple arrows shows the location of 10 IS elements present in *Francisella philomiragia subsp. philomiragia*. The black arrows show the location of 4rRNAs present in *Francisella noatunensis subsp. orientalis Toba04*.

A hallmark between sequenced *Francisella* genomes is a high degree of genomic rearrangements. These rearrangements are believed to be created by IS-elements and the elements are typically flanked by pseudogenes [8-10]. In addition, it has been reported that rearrangements may have been created by recombination near rRNAs [14]. Comparing *F. noatunensis subsp*. *orientalis Toba04* with the *F. philomiragia subsp. philomiragia* genome revealed several rearrangements (Figure 2) but there are no IS elements present in *F. noatunensis subsp. orientalis Toba04*. Even in the recently published paper where they have sequenced another *F. noatunensis subsp. orientalis* strain found weak evidence of a single IS element [4]. This opens for several different scenarios that could create the observed rearrangements seen in the tilapia parasite. One possibility is that the rearrangements are created by recombination near the rRNA genes. However, only 3 among the 4 rRNA genes in *F. noatunensis subsp. orientalis Toba04* are close to the breakpoint of just one block indicating that these rRNAs are not responsible for the other rearrangements observed between the two species (Figure 2). Another possibility is that the fish parasitic *Francisella* subspecies have had IS elements previously but have lost them and that the rearrangements have taken place prior to the loss of the IS elements. If the parasitic life-style in the fish infecting *Francisella* species is ancient this could also explain the lack of IS-elements in the genome (see above).

### The pseudogenes of *F. noatunensis* subsp. *orientalis Toba04 are old*

**Figure 3:**
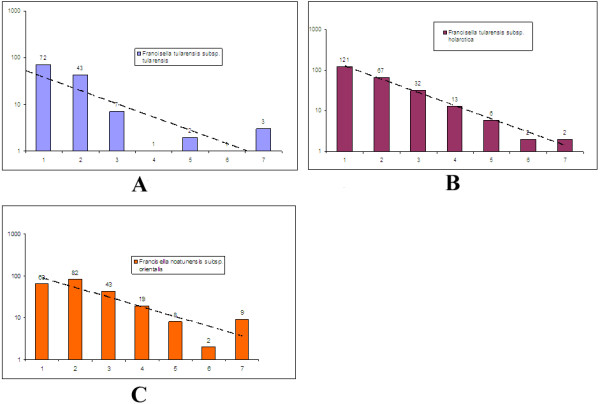
**The graphs show the number of pseudogenes with different number of inactivating mutations (x-axis).** Note that the Y-axis is logarithmic. **A**) This graph is generated using pseudogenes from *Francisella tularensis subsp. tularensis SCHUs4*. **B**) This graph is generated using pseudogenes from *Francisella tularensis subsp. holarctica OSU18*. **C**) This graph is generated using pseudogenes from *Francisella noatunensis subsp. orientalis Toba04*.

We identified 252 putative pseudogenes in the *F. noatunensis subsp*. *orientalis Toba04*, a number comparable with the other pathogenic *Francisella* species (see Table 1). We have manually curated the pseudogenes using the Artemis tool [15] aiming to avoid problems due to mutation error in homopolymer tracts often observed on pyrosequencing data. Since rearrangement is believed to be a major mechanism for generating pseudogenes in *Francisella* species one would expect that pseudogenes in *F. noatunensis subsp. orientalis Toba04* then would be older compared to those in *F. tularensis* strains which is still under-going genomic rearrangement and genome decay. In order to access the age of the pseudogenes in the sequenced *Francisella* genomes we counted the number of inactivating mutations in the different species. The analysis shows that *F. noatunensis subsp*. *orientalis Toba04* pseudogenes have significantly more inactivating mutations than the other pathogenic *Francisella* strains (Figure 3). If we assume a similar mutation rate in the different species, this indicates that the pseudogenes in *F. noatunensis subsp. orientalis Toba04* on average are older than the pseudogenes in the mammalian pathogenic *Francisella* strains (or alternatively posses a higher mutation rate). The high number of pseudogenes identified in the pathogenic *Francisella* species (see Table 1) is typical for bacteria associated with eukaryotic hosts [16]. It has been suggested that pseudogenes may be quickly removed from bacterial genomes (Kuo and Ochman 2010) and the majority of pseudogenes will only possess 1 inactivation mutation. The data obtained from the fish pathogenic *F. noatunensis subsp*. *orientalis Toba04* clearly demonstrate a higher proportion of pseudogenes with multiple inactivation mutations (see Figure 3) pointing towards older pseudogenes in this species.

### Old pseudogenes under neutral evolution in *F. noatunensis subsp. orientalis Toba04*

**Figure 4:**
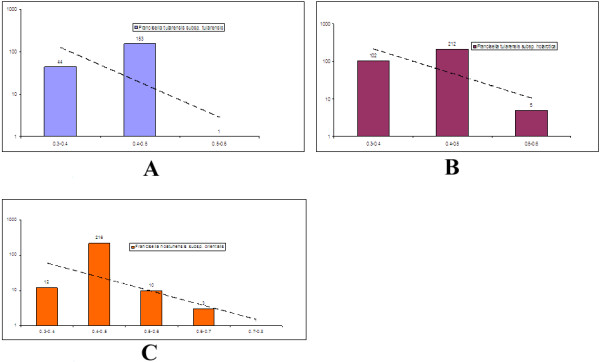
**The graphs show the number of pseudogenes (Y-axis) having different number of substitutions (X-axis).** Note that the Y-axis is logarithmic. The graphs refer to pseudogenes in Francisella tularensis subsp. tularensis SCHUs4 (**A**) Francisella tularensis subsp. holarctica OSU18 (**B**), and Francisella noatunensis subsp. orientalis Toba04 (**C**).

Several recent papers have addressed the extinction dynamics of pseudogenes in bacterial pathogens. Kuo and Ochman found that in *Salmonella*, there appears to be positive selection for deletion events leading to rapid removal of pseudogenes [17]. We performed two analyses with the pseudogenes present in *F. noatunensis subsp*. *orientalis Toba 04*, *F. tularensis subsp*. *tularensis SCHUs4* and *F. tularensis subsp*. *holarctica OSU18* to investigate the frequency and nature of pseudogenes in these genomes. For every pseudogene we counted the number of inactivating mutations and also calculated the nucleotide substitution rate between the pseudogenes and homologous gene. Given that the pseudogenes are not under selection (neutral evolution), one would expect a linear line in the log-normal graph. To some approximation, this is what we observe for all strains expect for *F. noatunensis subsp*. *orientalis Toba04* where we see fewer than expected pseudogenes with one inactivating mutation and also a larger than expected number with many (7 or more) inactivating mutations (Figure 3). The reason for this deviation may be that *F. noatunensis subsp*. *orientalis Toba04* has evolved more extensively after its transformation into an intracellular pathogen so that most pseudogenisation events are old. This is also consistent with old genomic rearrangements (happening before the IS elements were lost) since pseudogenisation is often a consequence of rearrangements. It may also be that *Francisella noatunensis subsp. orientalis Toba04* already lost all its non-essential genes some time ago so that losses of additional genes are deleterious. This would be consistent with the data for the other strains analysed if these have become pathogenic (and metabolically incompetent) more recently and the process of genome decay is still on-going. In the nucleotide substitution rate calculation analysis we found large number of pseudogenes at the rate of 0.4-0.5 (Figure 4) for *F. noatunensis subsp*. *orientalis Toba04*. This is also in agreement with the suggestion that the pseudogenisation has happened a long time ago in the Tilapia parasite. In none of the strains analyzed we see the pattern reported by Kuo and Ochman for *Francisella* indicating positive selection for pseudogenes deletion. This may relate to the difference in lifestyle between *Salmonella* and *Francisella* pathogens. The recent publication from Sjödin et al., also showed that *Francisella noatunensis* subspecies has evolved well before *Francisella tularensis* subspecies and *Francisella noatunensis subsp. orientalis* has no IS elements and the same scenario might have appeared in those strains as well [4].

### Comparative analysis of *F noatunensis subsp. orientalis Toba04* with other available *Francisella* genomes

**Figure 5:**
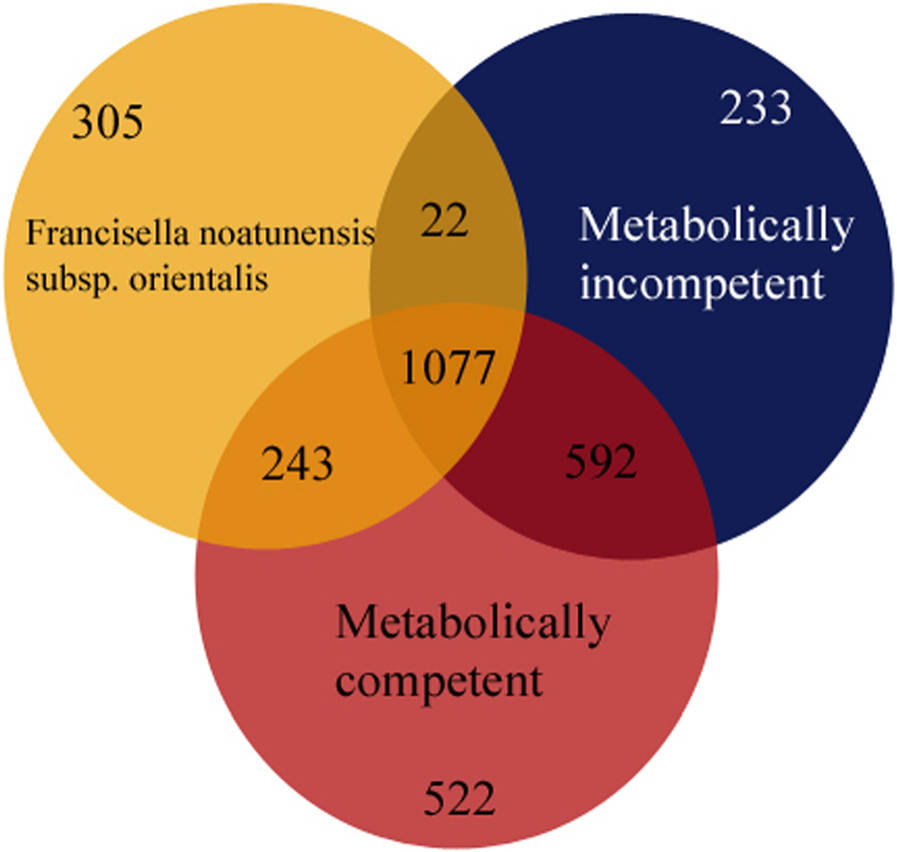
**The Venn diagram shows the number of genes (ortholog clusters) found to be shared between *F. noatunensis subsp. orientalis Toba04*, metabolically incompetent, and metabolically competent**.

We performed a comparison between the *Francisella* genomes (Table 1) in order to find shared and unique genes for each of the genomes. For this analysis we grouped similar genes shared between the species into ortholog clusters and the gene numbers given below refer to the number of ortholog clusters. We identified a set of 1,077 genes present in all the analyzed genomes – representing a potential core set of genes for *Francisella* (Figure 5). As expected, the free-living metabolically competent strains had the highest number of unique genes although the fish and mammalian parasites each have 305 and 233 unique genes among the *Francisella* genomes analyzed. This indicates that the pathogenic strains not only have lost genes after turning parasitic but they have also acquired genes. Whether gene loss or acquisition has happened before or after this transition cannot be ruled out from the available data. Gene acquisition is likely to happen by horizontal gene transfer. For 42 genes unique to *F. noatunensis subsp. orientalis Toba04* and for 30 genes unique to *F. tularensis subsp. tularensis* we found significant homology (more than 30% identity over at least 50% of the gene length) to genes in Genbank from bacteria species likely to be present in the same environment as the respective *Francisella* strains (Additional file 1: Tables S1 and S2). It is not unlikely that most of the other acquired genes in each of these pathogenic strains may be a result of horizontal gene transfer.

### Intact unique genes present in free-living *Francisella* genomes

We identified 522 genes present only in the metabolically competent genomes. The assumption is that these genes have been present also in the ancestors of the analyzed pathogenic strains but lost after they became parasites. In agreement with previous reports, we find that many of the 522 genes are involved in metabolism, intracellular transport and amino acid biosynthesis [8-10]. In addition we identified 17 membrane-associated proteins and 62 proteins annotated with signal-peptide (3 also annotated as membrane associated) which might play an important role in immunity during host parasite interactions, which are present only in metabolically competent genomes. Genes like the capsule polysaccharide biosynthesis protein required for immunity to destroy foreign antigens are present only in the metabolically competent genomes. We used DAVID [18] to identify over-represented functional terms (including Gene Ontology terms) among the unique genes from the metabolically competent species(compared to their frequency in the complete *Francisella philomiragia* genome). Functional terms related to DNA (integration, replication, binding, and metabolism), amino acid metabolism, transporters, membrane-association and signal peptides are among the most significant terms (Additional file 1: Figure S3). The present analysis narrows down the list of genes unique to free-living *Francisella*. Taken together, these results are consistent with earlier analyses and indicate that pathogenic *Francisella* strains have lost a substantial number of genes (512), and many of these have functions related to metabolism and DNA. This indicates that the parasitic *Francisella* species share many features in their adaptation to a parasitic lifestyle and that the fish pathogen may serve as a valuable model to study features of the mammalian parasitic *Francisella*.

### Intact unique genes present in the fish parasite *F. noatunensis subsp. orientalisToba04*

We found 305 genes unique for the *F. noatunensis subsp*. *orientalis* genome. Performing BLASTp search against non-redundant protein database we were able find significant matches for 260 genes which are not present in any of the other *Francisella* strains. Among these genes a putative lipopolysaccharide biosynthesis membrane protein is present. It is important for the virulent causing serious diseases in human and animals [19,20]. The other genes represent transporters, transferases, purine biosynthesis, thiamine biosynthesis, oxidation reduction, catalytic activities and hypothetical proteins and could be important for the pathogenecity of the strain.

### Intact unique genes present in the human parasitic *Francisella* genomes

The human parasitic *Francisella* genomes contain 233 genes that are not present in the other *Francisella* genomes studied. We compared these 233 genes with all genes in the non-redundant protein database using BLAST to find homologs in other mammalian strains and found 162 unique genes that are shared between *F. tularensis subsp. tularensis, F. tularensis subsp. holarctica*, and *F. tularensis subsp. mediasiatica* (Additional file 1: Table S3). There are 63 genes which are present only in *F. tularensis subsp. holarctica* strain, all of them hypothetical proteins. In *F. tularensis subsp. mediasiatica* 11 genes are unique and includes a protein involved in the type III restriction-modification system (FTM_0875), part of the defence mechanism against foreign DNA [21], where as *F. tularensis subsp. tularensis* has 44 unique genes and all of them hypothetical proteins. The unique proteins present in this strain might be related to the highly virulent nature in this strain although functions of these proteins are unknown. The other 44 genes are shared between these three strains. 30 of these genes have homologs in other bacteria and their presence in the *Francisella* genomes may be due to horizontal gene transfer. These genes represent functions like ATP binding, transferase activities and lipopolysaccharides biosynthesis and 11 of them are hypothetical proteins.

The genomes of the studied fish and human pathogenic genomes all contain genes unique among *Francisella* species. It seems unlikely that they have all been present in an ancestral *Francisella* genome and more likely that some genes have been horizontally transferred from other organisms, likely in most cases from environmental bacteria. Most of the genes like lipid biosynthesis, polysaccharides biosynthesis, cold-shock DNA-binding domain-containing protein, membrane protein and other genes in the horizontal gene transfer list could have been useful for survival of the pathogen or be involved in virulence. The hypothetical proteins present in *F. tularensis subsp. tularensis* could be important to determine the high virulent nature of this species. Having a novel pathogenic *Francisella* genomic sequence available, we use the opportunity to analyze in a comparative manner with the selected set of *Francisella* genomes (Table 1) with focus on systems that are believed to be important for virulence. The distant evolutionary relationship between the human pathogenic and the fish pathogenic strains, can potentially give some insight into virulence and also genome decay and what is the essential, core, set of genes in *Francisella*.

### Virulence mechanism

#### Francisella Pathogenecity Island and its role in virulence

Most of the intracellular pathogen’s virulence mechanisms are activated by type III or type IV secretion system [22]. However, in *Francisella* species the genes involved in Pathogenecity Island is related to type VI secretion system [9,10]. The genomes of the human parasitic *Francisella* species possess two copies of FPIs where as *F. noatunensis subsp*. *orientalis Toba04* and the free-living, metabolically competent species, only contain one copy. The human parasites have likely acquired an extra copy of the FPI after diverging from the free-living relatives [11]. The FPI consists of 17 genes.

We compared the FPI regions between the human pathogenic, the free-living, and the fish pathogenic *F. noatunensis subsp. orientalis Toba04* genome (Table 1); we found that pdpD is missing only from the *F. tularensis subsp. holarctica* genome. Previous studies report that replacement or loss of pdpD reduced the expression of iglA [23]. It may be a factor for the less virulent nature of these genomes in human. We also found that the pdpC gene is missing in *F. noatunensis subsp. orientalis Toba04* and *F. philomiragia* subsp. *philomiragia,* but present in all other genomes analyzed. It has been suggested that pdpC is essential for infection in mammalian cells [24]. The absence in *F. noatunensis* subsp. *orientalis Toba04* suggests that pdpC is not essential for *Francisella* to infect fish. In agreement with the phylogenetic analyses presented earlier, most of the amino acid sequences of the proteins encoded in the FPI region of *F. noatunensis* subsp. *orientalis* are more similar to the corresponding genes in *F. philomiragia* subsp*. philomiragia* than to those in the human pathogenic genomes analyzed.

#### Oxidative stress response

Oxidative stress response plays a major role in virulence. The LysR family of regulatory proteins are regulators for oxidative stress response [10,25]. In the *F. noatunensis subsp. orientalis Toba04* genome there are 6 genes encoding for LysR protein family regulators (OOM_0025, OOM_0069, OOM_0378, OOM_0457, OOM_1159, OOM_1654). There are 8 LysR proteins present in *F. tularensis subsp. tularensis SCHUs4* and 5 LysR proteins in *F. tularensis* subsp. *holarctica OSU18*. Interestingly LysR proteins are not found in *F. tularensis* subsp. *mediasiatica* strains and the absence of LysR proteins in *F. tularensis* subsp*. mediasiatica* could be a factor explaining their moderate virulence.

#### Secretion system

Bacterial pathogenesis is fundamentally regulated by secretion systems playing a role in transferring the virulence factors through the cell wall from the pathogen to the host. Previous papers report that components of Twin arginine translocation (TAT), Type I, Type II, Type V, and Type VI secretion system, have been found in *F. tularensis* subsp. *tularensis*[26-28]. Searching the genome for T3SS components, resulted in identification of 7 predicted proteins which have components of the T3SS effectors (Table 2). Among them are methionine aminopeptidase and haloacid dehalogenase-like hydrolase (OOM_1052, OOM_1036 which previously are reported from *F.tularensis subsp*. *tularensis SCHUs4*[10]. Despite analogous analyses, we were not able to identify homologs for type IV secretion system proteins in the *F. noatunensis subsp*. *orientalis Toba04* genome.

**Table 2:** **The type 3 secretion type proteins present in *Francisella noatunensis *subsp.*orientalis***

***F.noatunensis subsp. orientalisToba04***	**Protein**	**Homologous T3SS effector’s NCBI geneID**
**ID**		
OOM_1477	phosphoribosylglycinamide synthetase	28868095
OOM_1473	phosphoribosylglycinamide synthetase	28868095
OOM_1080	glycogen branching enzyme	8714177
OOM_1052	methionine aminopeptidase	12512890
OOM_1036	haloacid dehalogenase-like hydrolase	28868062
OOM_0422	hypothetical protein	34497721
OOM_0090	pyrimidine reductase/pyrimidine deaminase	28868061

Type 4 pili proteins in bacteria are generally involved in motility. These proteins play a major role in bacterial virulence since they facilitate entrance of the bacteria into the host [29] and several have been reported to be present in *Francisella* species [9-11]. There are 6 type iv pili proteins present in the *F. noatunensis subsp. orientalis Toba04* genome (OOM_0045, OOM_0401, OOM_0402, OOM_0611, OOM_1408, OOM_1374). We also note that the pilA gene represented by three ORFs in *F. tularensis subsp. tularensis* (FTT_0888, FTT_0889, FTT_0890) and one ORF (FTN0413) in *F. tularensis subsp. novicida* important for mediating virulence [30] is absent in *F. noatunensis subsp. orientalis*. A PilA gene family protein (OOM_1408) with different amino acid sequence is present only in *F. noatunensis subsp. orientalis Toba04* and *F. philomiragia subsp. philomiragia*.

#### Two-component regulatory system

Two-component regulatory systems are important for recognition of environmental changes and virulence in bacterial pathogens [31,32]. The kdpD gene belonging to the two component regulatory system [10] is present in *F. tularensis subsp*. *tularensis, F. tularensis subsp. mediasiatica, F. tularensis subsp. novicida, F. philomiragia subsp. philomiragia* but absent in *F. noatunensis subsp. orientalis Toba04 and F. tularensis subsp. holarctica*. In addition, we found that the two-component regulatory sensor histidine kinase (Fphi_1001) gene is present only in metabolically competent *Francisella* species and not present in all the metabolically incompetent proteins including *F. noatunensis subsp. orientalisToba04*. This protein is important for the stimulus response when any virulent species enters into the host organism.

#### Iron acquisition system

Iron acquisition is crucial as a virulence factor. Bacteria need iron inside the phagosomes for growth and iron deficiency leads to abnormal cell development [25,33]. Ferric uptake regulatory protein (FTT_0030) is modulating the iron uptake system in *F. tularensis subsp. tularensis*[9], and no other gene has been found in this genome regulating iron content. Two proteins IucA/IucC (OOM_0522) and Ferrous iron transporter (OOM_0685) involved in siderophore synthesis and iron transport are present only in *F. noatunensis subsp. orientalis Toba04* and in the metabolically competent genomes. Siderophore synthesis is one of the major mechanisms for iron aquisition in fish. Absence of genes required for siderophore synthesis in *F. tularensis* subspecies shows a different host adaptation for human virulent *Francisella* subspecies.

#### Comparison of metabolic pathways from *F. philomiragia subsp. philomiragia ATCC 25017*, *F. noatunensis subsp. orientalis Toba04 and F. tularensis subsp. tularensis SCHUs4*

A metabolic network for *F.tularensis subsp. tularensis SCHUs4* was published together with its genome [9]. We performed computational prediction of the metabolic pathways for the *F. philomiragia subsp. philomiragia* using *F.tularensis subsp. tularensis SCHUs4* as reference model. Then we used *F. philomiragia subsp. philomiragia* and *F. tularensis subsp. tularensis SCHUs4 as* reference to predict the corresponding pathways for *F. noatunensis subsp*. *orientalis Toba04*. In *F. noatunensis subsp. orientalis Toba04,* we predicted 798 enzymes involved in small molecule metabolism in a total of 1099 enzymatic reactions. In addition, 201 small molecule metabolic pathways were predicted from the genome data. Not all pathways are complete since for some of the reactions the enzymes involved have not been found in the genome. Our analysis identifies 329 such ”pathway holes” and none of these could be filled using pathway tools software (see Methods). The large number of pathway holes indicates many disrupted pathways and is consistent with genome decay often found in intracellular pathogenic bacteria (Table 3).

**Table 3:** The important amino acid pathways required for the growth of Francisella subspecies are given

**Amino acid biosynthesis pathways required for growth**	***F. tularensis subsp. tularensis SCHUs4***	***F. philomiragia subsp. philomiragiaATCC 25017***	***F. noatunensis subsp. orientalisToba04***
Asparagine	1/1	1/1	no
Cysteine	2/2	2/2	2/2
Serine	2/3	2/3	2/3
Threonine	1/2	2/2	2/2
Methionine	no	1/4	1/4
Tyrosine	1/3	2/3	1/3
Lysine	3/10	7/10	4/10
Proline	2/4	4/4	3/4
Arginine	no	4/4	4/4
Histidine	no	7/10	no
Valine	3/4	4/4	3/4
Iso-leucine	4/5	4/5	3/5
Leucine	5/9	9/9	6/9

The values represent the number of enzymes present compared to the total number of enzymes needed.

There are 14 amino acid pathways essential for the growth of the *Francisella tularensis subspecies* (Asp, Cys, Ser, Thr, Met, Tyr, Lys, Pro, Arg, His, Val, Ile, and Leu) [34]. In addition, the *F. tularensis* subsp. *tularensis SCHUs4* pathways for sulphate assimilation, threonine biosynthesis, valine biosynthesis and isoleucine biosynthesis are incomplete together with pathways for methionine, arginine, histidine, lysine and tyrosine biosynthesis in the same subspecies [9]. However, in our computational prediction of pathways in *F. tularensis subsp. tularensis* suggests that it has pathways for synthesis of all amino acids except Arg and His. For *F.* noatunensis *subsp. orientalis Toba04* we are not able find the pathways for His, Asp and Cys.

Enzymes required for the His biosysnthesis pathway is only found in *F. philomiragia* subsp*. philomiragia*. In *F. noatunensis* subsp. *orientalis*, the genes required for His biosynthesis are present as pseudogenes. The pathway is also absent in *F. tularensis* subsp. *tularensis SCHUs4*. It is of interest to note the absence of the pathway for Asp synthesis. Asparagine is an essential amino acid in fish specific *F. noatunensis* subsp. *orientalis Toba04*, suggesting that this amino acid may be taken from the host. We were able to find complete pathways for Asp, Cys, Thr, Pro, Arg, Val and Leu in *F. philomiragia* subsp. *philomiragia* indicating that pathways for synthesizing all these amino acids were present in the ancestral *Francisella* genome and lost in the metabolically incompetent genomes. The pathway for Sulfate assimilation is absent in *F. tularensis* subsp*. tularensis SCHUs4* and present in *F. philomiragia* subsp*. philomiragia* (Additional file 1: Figure S4).

## Conclusions

We have presented the whole genome characterisation of *F. noatunensis subsp*. *orientalis Toba04* and extensive comparative analysis against other *Francisella* subspecies. All the *Francisella* strains that are non-virulent to human possess one set of Pathogenecity Island and very low number of IS elements. *F. noatunensis subsp. orientals Toba04* which is most closely related to *F. philomiragia subsp. philomiragia* has no IS elements present in its genome. IS elements are important for generating genomic rearrangements typically observed between *Francisella* species. Since the *F. noatunensis subsp. orientalis Toba04* genome is significantly rearranged compared to other *Francisella* species we propose that IS elements have been present but they are now lost. In addition, we identified 252 pseudogenes in *F. noatunensis subsp. orientalis* and they are typically created as a result of genomic rearrangements. The analysis of the pseudogenes from *Francisella* species demonstrated that the pseudogenes from *F. noatunensis subsp*. *orientalis* are old by having more than one inactivating mutation*.* The whole genome phylogenetic analysis revealed two main branches that separate the mammalian and fish parasitic *Francisella* species.

Although the pathogenic *Francisella* species resides on different phylogenetic branches they share a set of common features like a large number of pseudogenes and several interrupted metabolic pathways resulting in metabolic incompetence. The metabolic incompetence is like an adaptation to an intracellular life style and points to similar evolutionary constraints from the different vertebrate hosts.

Our work provides insight into studies of *Francisella* subspecies evolution, and our comparative analysis and results will help to understand the pathogenecity mechanisms for each *Francisella* subspecies. We have also listed important genes influencing the virulent mechanisms in each pathogenic strain specifically so that researchers working on *Francisella* could work on those genes for further understanding on virulent factors. In addition, we found both fish and human pathogens share many features and it may be possible to use the fish parasites as models to enhance our knowledge about host parasite interactions for this important group of pathogens.

## Methods

### Sequencing and assembly

*The F. noatunensis subsp. orientalis Toba04* strain was sequenced using 454-pyrosequencing [35] generating 263,717 reads consisting of 56,522,682 bases. These were assembled using Newbler v.2 [35]http://454.com/products-solutions/analysis-tools/gs-de-novo-assembler.asp leading to 21 contigs with total length of 1,848,209 bases and N50 of 215,480 base pairs. The gaps were closed by first analysing the contig graph, which presents the connections between contigs, based on the repeat information present in the reads. The edges between the contigs with coverage less than 10 were removed. Further comparative analysis (using BLAST) with fully sequenced strains of *F.tularensis* subsp. *tularensis SCHUs4* was performed, and the edges between contigs which were inconsistent across these strains were removed and incremental assembly using runViewer program present in the Newbler was performed. This led to 8 contigs with total length of 1,847,034 and N50 of 429,132. Later a series of PCR, suggested on the basis of contig graph, were performed to join these contigs and check the correctness of assembly. Several sets of specific primers were designed at the ends of each contig.

GoTaq PCR enzyme (Promega) was used in all amplifications. Two and two primers were combined, in individual PCR reactions, so that all possible combinations of primers were tested (an all against all combination approach). Genomic DNA identical to the DNA used for sequencing was used as template. Fragments appearing as single bands in agarose gel electrophoresis were either purified using ExoSAP-IT (GE Healthcare) and sequenced on both strands using the PCR primers and the BigDye chemistry (Applied Biosystems), or the PCR fragments were cloned into a pCR4_TOPO vector following the supplied instructions (Invitrogen) and several random clones were sequenced using the vector primers. All the sequences were used in the assembly. To check the correctness of assembly 10 pair of primers were designed in one of the contigs and PCR reactions were performed. All reactions gave result as expected. This led to 19 primers out of which 12 gave products (5 were one sided) and 7 failed. This information was fed back into the assembler as fake paired ends and another series of incremental assembly was performed leading to 5 contigs with 1,715,028 bases and N50 of 1,033,009. Another series of PCR based on these results led to 63 primer products and information about repeats and orientation. A straight-forward fake paired-end presentation into the assembler broke up the assembly due to short matches of product in various locations confusing the assembler. So the reassembly was done incrementally in a fashion maintaining the consistency between PCR results and reads based assembly. This lead to 1 big (length 1,857,341) and 3 small scaffolds (total length 8940). The pink line in Figure 5 is the scaffold linking while plain black line contig linking. Since all the contigs do not have paired end connection information, they don’t show in the scaffold-graph (including contigs of less than 500 bases), these gaps can also be filled manually (just replacing the gap with contigs based on graph). For example between 14 and 15 we can put in 3, 2, and 5 but Newbler did not have enough information to do it automatically (Additional file 1: Figure S2).

### Annotation

We used the prokaryotic annotation pipeline from TIGR to annotate the genome: Glimmer 3 [36] was used to predict the genes in the genome. NcRNAdb [37] and RNammer [38] was used to predict 23s and 16s RNA genes. tRNAscan-SE [39] was used to predict tRNAs present in the genome. We used TMHMM [40] to predict transmembrane helices and SignalP [41] to predict signal peptides. The ORFs predicted were compared against NCBI’s non-redundant database using BlastX. Protein coding genes from predicted results were curated manually using Artemis [15]. All the predicted proteins were searched against NCBI’s COG database (Clusters of Orthologous Groups of proteins based on phylogenetic classification of proteins encoded in complete genomes) to find the protein family. Cognitor was used to predict the COGs for each protein [42]. EC numbers were assigned to the proteins using the BRENDA database [43]. Interpro-Scan was used to add domain based annotation of the proteins [44]. CGview was used to make circular genome plot [45].

### IS elements and genome rearrangement plot

IS-finder [46] web server was used to find the IS-.elements. We used BLASTx [47] to compare against the IS elements database and the results were manually checked. Nucmer in Mummer 3.0 [48] package was used to prepare the comparison plot between *F. noatunensis subsp. orientalis* and *F. philomiragia subsp. philomiragia*. To plot *F. noatunensis subsp. orientalis’s* pseudogenes against *F. philomiragia* subsp.*philomiragia* genome, we compared pseudogenes against *F. philomiragia subspecies* genome and the gene coordinates were extracted from BLASTp results using in-house Perl program. We used that coordinates to map the pseudogenes in the comparison plot.

### Mutation count

To count the number of mutation in the pseudogenes in *Francisella subspecies*, we compared all the pseudogenes present in the genomes against the NCBI’s non-redundant database. From the Blast result we calculated the possible inactivating mutations including insertions, substitutions, premature stop codons and point mutations using a Perl program written in-house. The log-normal graphs were made using MS-Excel.

### Maximum likelihood distance

We calculated the maximum likelihood distance between the pseudogenes present in *Francisella species* and its 1^st^ homologous hit from BLASTp result. We used biodist module in bio++ package to calculate the maximum likelihood distance values using L95 nucleotide substitution model.

### Phylogenetic tree

Mauve 2.3.1 [49] was used to prepare the whole genome alignment between all the *Francisella species*. The same software was also used to show the rearrangement between *F. noatunensis subsp*. *orientalis* and *F. philomiragia subsp. philomiragia*. The location of rRNAs and IS-elements were marked manually. The genome alignment was loaded in to the MEGA4 [50] for editing and subsequently the Neighbor-joining method was used to predict the tree with 1000 replicates for the bootstrap value calculation. We used the same procedure for the phylogenetic tree predicted using core gene sets. Incomplete assembly of *F. noatunensis subsp. noatunensis* was compared against protein sequences of *F. noatunensis subsp. orientalis* to find core set of genes commonly present in *Francisella species*.

### Metabolic pathways

Pathway tools software [51] was used to predict the metabolic pathway for *F. noatunensis subsp. orientalis*. We also predicted metabolic pathway for *F. philomiragia subsp. philomiragia* to use it as reference for *F. noatunensis subsp. orientalis* and comparative analysis. The Pathologic module was used to predict the pathways for both the genomes. The function Overviews->highlight->species comparison were used to compare pathways between genomes.

### Genome comparison

Protein sequences from *F. tularensis subsp. tularensis SCHUs4*, *F. tularensis subsp*. *holarctica OSU18*, F*. tularensis subsp. mediasiatica FSC 147*, *F. tularensis subsp. novicida U112*, *F. philomiragia subsp. philomiragia ATCC 25017* and *F. noatunensis subsp. orientalis*genomes were extracted from GenBank file. We used BLASTp [47] to compare the protein sequences against themselves to find unique and the genes which are shared between the genomes. We calculated two values to classify if two proteins from different species are same: (i) we calculated the score bit percentage from the top homologous hit. We divided the top hit’s score bit value by the values from subsequent hits from other *Francisella* species. The score bit percentage should be >=65% of top homologous hit’s score bit to classify the same two proteins. (ii) We calculated the alignment length percentage between the query and subject. The alignment length percentage is calculated from the length of the subject protein and length of the alignment. It should be >=75% between query and subject to classify two proteins as the same. We used the same method to cluster the proteins in to ortholog groups.

## Competing interests

The authors declare that they have no competing interests.

## Authors’ contributions

IJ and FN were involved in planning the project and also in writing the manuscript; SS did the annotation, all analyses and wrote the draft manuscript, HK helped in closing the gaps in the genome assembly, AS assembled the genome. All authors read and approved the final manuscript.

## Supplementary Material

Additional file 1 **Table S1.**The table lists the unique proteins present in Francisella noatunensis subsp.orientalis Toba04 which might have transferred from other bacteria through horizontal gene transfer. Genes which has at least 50% alignment match to the original protein has been selected. **Table S2:** The table lists the unique proteins present in *Francisella tularensis* subspecies which might have transferred from other bacteria through horizontal gene transfer. Genes which has at least 50% alignment match to the original protein has been selected. **Table S3:** The table lists the proteins which are present uniquely in each *Francisella tularensis* subspecies. Where as in the subspecies column “H” means the protein is present in *Francisella tularensis subsp*. *holarcticaOSU18*, “M” means the protein is present in *Francisella tularensis subsp*. *mediasiaticaFSC 147,* and “T” means the protein is present in *Francisella tularensis subsp*. *tularensis SCHU s4*. Entries with multiple letters are present in multiple subspecies. **Figure S1.** Circular Genome view of *Francisella noatunensis subsp.orientalisToba04.***Figure **2. Map showing links between contigs. **Figure **3. Result from DAVID server showing over representation of functional terms in metabolic competent species. **Figure **4. The given figure is one part of the comparison of *F. noatunensis subsp. orientalis Toba04* and *F. philomiragia subsp. philomiragia ATCC 25017* metabolic pathway. Red colour indicates the reaction is shared between *F. noatunensis subsp. orientalis* and *F. philomiragia* subsp*. philomiragia*. Thin green lines indicates pathway hole in *F. philomiragia subsp. philomiragia.* Thick green line is the presence of reaction in *F. philomiragia subsp. philomiragia.* Click here for file
