# Supplementary material for: Whole genome sequencing of the fish pathogen Francisella noatunensis subsp. orientalis Toba04 gives novel insights into Francisella evolution and pathogenecity
Source: BMC Genomics. 2012 Nov 6;13:598. doi: 10.1186/1471-2164-13-598 (PMC3532336; doi:10.1186/1471-2164-13-598)
Supplement: Additional file 1 — Table S1.The table lists the unique proteins present in Francisella noatunensis subsp.orientalis Toba04 which might have transferred from other bacteria through horizontal gene transfer. Genes which has at least 50% alignment match to the original protein has been selected. Table S2: The table lists the unique proteins present in Francisella tularensis subspecies which might have transferred from other bacteria through horizontal gene transfer. Genes which has at least 50% alignment match to the original protein has been selected. Table S3: The table lists the proteins which are present uniquely in each Francisella tularensis subspecies. Where as in the subspecies column “H” means the protein is present in Francisella tularensis subsp. holarcticaOSU18, “M” means the protein is present in Francisella tularensis subsp. mediasiaticaFSC 147, and “T” means the protein is present in Francisella tularensis subsp. tularensis SCHU s4. Entries with multiple letters are present in multiple subspecies. Figure S1. Circular Genome view of Francisella noatunensis subsp.orientalisToba04.Figure 2. Map showing links between contigs. Figure 3. Result from DAVID server showing over representation of functional terms in metabolic competent species. Figure 4. The given figure is one part of the comparison of F. noatunensis subsp. orientalis Toba04 and F. philomiragia subsp. philomiragia ATCC 25017 metabolic pathway. Red colour indicates the reaction is shared between F. noatunensis subsp. orientalis and F. philomiragia subsp. philomiragia. Thin green lines indicates pathway hole in F. philomiragia subsp. philomiragia. Thick green line is the presence of reaction in F. philomiragia subsp. philomiragia. [file 1471-2164-13-598-S1.doc]

***Whole genome sequencing of the fish pathogen Francisella noatuensis subsp. oriental gives novel insights into Francisella evolution and pathogenicity***

Settu Sridhar, Animesh Sharma, Heidi Kongshaug, Frank Nilsen and Inge Jonassen.

**Supplementary material**

Supplementary Table 1 (S1):

The table lists the unique proteins present in Francisella noatunensissubsp.orientalis which might have transferred from other bacteria through horizontal gene transfer. Genes which has at least 50% alignment match to the original protein has been selected.

| **Locus_ID** | **Original Seq_length** | **Alignmnet_**  **length** | **Alignment_**  **percentage** | **Function** | **Species** |
| --- | --- | --- | --- | --- | --- |
| OOM_0819 | 248 | 124 | 50 | acetyltransferase, GNAT family protein | Bacillus sp. NRRL B-14911 |
| OOM_0833 | 441 | 257 | 58 | ABC transporter-like protein | Sulfurimonas autotrophica DSM 16294 |
| OOM_1473 | 414 | 244 | 59 | biotin carboxylase | Burkholderia pseudomallei 668 |
| OOM_0823 | 336 | 203 | 60 | glycosyl transferase | Helicobacter bilis ATCC 43879 |
| OOM_1625 | 377 | 234 | 62 | group 1 glycosyl transferase | Desulfobulbus propionicus DSM 2032 |
| OOM_1467 | 215 | 137 | 64 | 4'-phosphopantetheinyl transferase | Burkholderia multivorans ATCC 17616 |
| OOM_0355 | 154 | 99 | 64 | regulatory protein for C-P lyase | Stigmatella aurantiaca DW4/3-1 |
| OOM_1464 | 330 | 225 | 68 | hypothetical protein | Vibrionales bacterium SWAT-3 |
| OOM_1775 | 154 | 107 | 69 | snoaL-like polyketide cyclase family protein 2 | Achromobacter xylosoxidans A8 |
| OOM_0851 | 185 | 129 | 70 | conserved hypothetical protein | Clostridium hathewayi DSM 13479 |
| OOM_0669 | 314 | 219 | 70 | putative acyltransferase | Moritella sp. PE36 |
| OOM_1387 | 437 | 307 | 70 | 4-hydroxybutyrate coenzyme A transferase | Legionella longbeachae D-4968 |
| OOM_0828 | 354 | 264 | 75 | glycosyl transferase family protein | Pectobacterium carotovorum subsp. brasiliensis PBR1692 |
| OOM_1614 | 208 | 172 | 83 | hypothetical protein | Methanospirillum hungatei JF-1 |
| OOM_1612 | 330 | 283 | 86 | UDP-GlcNAc-specific C4,6 dehydratase/C5 epimerase | Campylobacterales bacterium GD 1 |
| OOM_0200 | 165 | 145 | 88 | urease accessory protein UreD | Arcobacter butzleri RM4018 |
| OOM_1616 | 344 | 304 | 88 | pseudaminic acid synthase | Denitrovibrio acetiphilus DSM 12809 |
| OOM_0817 | 79 | 71 | 90 | acyl carrier protein | Candidatus Methanoregula boonei 6A8 |
| OOM_1707 | 137 | 124 | 91 | glyoxalase | Arcobacter butzleri JV22 |
| OOM_1752 | 387 | 351 | 91 | hypothetical protein | Bacillus atrophaeus 1942 |
| OOM_1619 | 312 | 283 | 91 | hypothetical protein | Acaryochloris marina MBIC11017 |
| OOM_1470 | 321 | 297 | 93 | hypothetical protein | Bradyrhizobium sp. BTAi1 |
| OOM_0821 | 524 | 488 | 93 | amino acid adenyltransferase | Streptomyces clavuligerus ATCC 27064 |
| OOM_0807 | 221 | 208 | 94 | acyltransferase 3 | Pseudomonas brassicacearum subsp. brassicacearum NFM421 |
| OOM_1161 | 224 | 212 | 95 | 12-oxophytodienoate reductase | Fluviicola taffensis DSM 16823 |
| OOM_0366 | 273 | 260 | 95 | phosphomethylpyrimidine kinase | Legionella drancourtii LLAP12 |
| OOM_0367 | 220 | 210 | 95 | TenA family transcriptional regulator | Brachyspira murdochii DSM 12563 |
| OOM_0365 | 312 | 299 | 96 | thiamine-monophosphate kinase | Alistipes shahii WAL 8301 |
| OOM_1390 | 172 | 165 | 96 | MoaC domain protein | Legionella longbeachae D-4968 |
| OOM_0818 | 253 | 244 | 96 | 3-ketoacyl-(acyl-carrier-protein) reductase | Hahella chejuensis KCTC 2396 |
| OOM_0826 | 152 | 147 | 97 | lipopolysaccharide biosynthesis protein | Providencia rettgeri DSM 1131 |
| OOM_1169 | 270 | 263 | 97 | alpha/beta hydrolase fold protein | Nostoc punctiforme PCC 73102 |
| OOM_1389 | 284 | 277 | 98 | citrase lyase beta subunit | Legionella longbeachae D-4968 |
| OOM_0363 | 212 | 208 | 98 | putative thiamine-phosphate pyrophosphorylase | Chlamydophila psittaci RD1 |
| OOM_1613 | 386 | 380 | 98 | pyridoxal phosphate-dependent enzyme | Acinetobacter baumannii ACICU |
| OOM_0364 | 262 | 258 | 98 | hydroxyethylthiazole kinase | Chlamydophila caviae GPIC |
| OOM_1615 | 232 | 229 | 99 | acylneuraminate cytidylyltransferase | Arcobacter butzleri RM4018 |
| OOM_0368 | 94 | 93 | 99 | XRE family transcriptional regulator | Legionella drancourtii LLAP12 |
| OOM_1620 | 307 | 304 | 99 | NAD-dependent epimerase/dehydratase | Mucilaginibacter paludis DSM 18603 |
| OOM_0357 | 155 | 154 | 99 | acetyltransferase | Legionella drancourtii LLAP12 |
| OOM_0358 | 273 | 272 | 100 | conserved hypothetical protein | Clostridium sp. 7_2_43FAA |
| OOM_0356 | 343 | 343 | 100 | acetyltransferase | Rickettsia bellii RML369-C |

Supplementary Table 2 (S2):

The table lists the unique proteins present in Francisella tularensis subspecies which might have transferred from other bacteria through horizontal gene transfer. Genes which has at least 50% alignment match to the original protein has been selected.

| **Locus_ID** | **Original Seq_length** | **Alignmnet_**  **length** | **Alignment_**  **percentage** | **Function** | **Species** |
| --- | --- | --- | --- | --- | --- |
| FTH_0336 | 180 | 90 | 50 | GNAT family acetyltransferase | Bacillus megaterium QM B1551 |
| FTT_0662c | 197 | 100 | 51 | hypothetical protein | Erythrobacter litoralis HTCC2594 |
| FTH_0807 | 183 | 95 | 52 | PRC-barrel | Nitrosospira multiformis ATCC 25196 |
| FTT_0401 | 423 | 224 | 53 | hypothetical protein | Arthrobacter arilaitensis Re117 |
| FTH_0755 | 654 | 351 | 54 | transglutaminase | Haliangium ochraceum DSM 14365 |
| FTH_0564 | 260 | 142 | 55 | hypothetical protein | Candidatus Accumulibacter |
| FTT_0487 | 412 | 226 | 55 | macrolide-efflux protein | Coxiella burnetii Dugway 5J108-111 |
| FTH_1212 | 301 | 166 | 55 | membrane protein | Vibrio sp. RC586 |
| FTH_1231 | 253 | 141 | 56 | hypothetical protein | Xanthomonas axonopodis pv. citri |
| FTT_1122c | 162 | 91 | 56 | putative lipoprotein | Aliivibrio salmonicida LFI1238 |
| FTT_0727 | 320 | 186 | 58 | secretion protein, HlyD family | Campylobacter showae RM3277 |
| FTH_0411 | 120 | 72 | 60 | hypothetical protein | Thiomicrospira crunogena XCL-2 |
| FTT_1172c | 193 | 116 | 60 | cold-shock DNA-binding domain-containing protein | Burkholderia multivorans ATCC 17616 |
| FTH_0719 | 1052 | 651 | 62 | ribonuclease E | Vibrio cholerae TM 11079-80 |
| FTM_0677 | 138 | 93 | 67 | transposase | Synechocystis sp. PCC 6803 |
| FTM_0934 | 206 | 139 | 67 | GDSL family lipase | Syntrophobacter fumaroxidans MPOB |
| FTT_0158c | 273 | 191 | 70 | hypothetical protein | Enterococcus faecalis |
| FTT_1646 | 145 | 103 | 71 | D-Tyr-tRNA(Tyr) deacylase; removes other D-aminoacids from mischarged | Pseudoalteromonas tunicata D2 |
| FTH_0955 | 771 | 559 | 73 | Type I restriction-modification system, M subunit, putative | Arcobacter butzleri RM4018 |
| FTT_0267 | 390 | 289 | 74 | hypothetical protein | Allochromatium vinosum DSM 180 |
| FTM_0878 | 318 | 252 | 79 | hypothetical protein | Photobacterium profundum SS9 |
| FTM_0875 | 991 | 820 | 83 | restriction enzyme of type III restriction-modification system | Bartonella tribocorum CIP 105476 |
| FTT_0496 | 253 | 214 | 85 | hypothetical protein | Fluviicola taffensis DSM 16823 |
| FTT_0888c | 147 | 127 | 86 | fimbrial protein | Neisseria polysaccharea ATCC 43768 |
| FTT_1068c | 196 | 186 | 95 | hypothetical protein | Sulfurimonas autotrophica DSM |
| FTH_0962 | 356 | 344 | 97 | putative ABC transporter ATP-binding protein YheS | Haemophilus parasuis 29755 |
| FTT_0677c | 255 | 248 | 97 | methyltransferase type 11 | Dickeya dadantii Ech703 |
| FTT_0254c | 59 | 58 | 98 | hypothetical protein gbs0364 | Streptococcus agalactiae NEM316 |
| FTM_0654 | 665 | 654 | 98 | transketolase | Oxalobacter formigenes OXCC13 |
| FTM_1557 | 277 | 276 | 100 | protein of unknown function with TPR repeat region and von Willebrand | Shewanella sediminis HAW-EB3 |

Supplementary Table 3 (S3):

The table lists the proteins which are present uniquely in each Francisella tularensis subspecies. Where as in the subspecies column “H” means the protein is present in Francisella tularensis subsp.holarctica, “M” means the protein is present in Francisella tularensis subsp.mediasiatica, and “T” means the protein is present in Francisella tularensis subsp.tularensis. Entries with multiple letters are present in multiple subspecies.

| **Locus_ID** | **Function** | **Subspecies** |
| --- | --- | --- |
| FTH_0047 | hypothetical protein | H |
| FTH_0055 | hypothetical protein | H |
| FTH_0135 | conserved hypothetical protein | H |
| FTH_0149 | hypothetical protein | H |
| FTH_0163 | hypothetical protein | H |
| FTH_0292 | hypothetical protein | H |
| FTH_0303 | conserved hypothetical protein | H |
| FTH_0381 | conserved hypothetical protein | H |
| FTH_0411 | conserved hypothetical protein | H |
| FTH_0439 | hypothetical protein | H |
| FTH_0443 | hypothetical protein | H |
| FTH_0445 | hypothetical protein | H |
| FTH_0465 | hypothetical protein | H |
| FTH_0466 | hypothetical protein | H |
| FTH_0467 | hypothetical protein | H |
| FTH_0523 | hypothetical protein | H |
| FTH_0525 | hypothetical protein | H |
| FTH_0564 | conserved hypothetical protein | H |
| FTH_0567 | hypothetical protein | H |
| FTH_0615 | hypothetical protein | H |
| FTH_0616 | hypothetical protein | H |
| FTH_0619 | hypothetical protein | H |
| FTH_0622 | hypothetical protein | H |
| FTH_0653 | hypothetical protein | H |
| FTH_0670 | hypothetical protein | H |
| FTH_0708 | hypothetical protein | H |
| FTH_0755 | possible transglutaminase | H |
| FTH_0785 | conserved hypothetical protein | H |
| FTH_0787 | hypothetical protein | H |
| FTH_0807 | conserved hypothetical protein | H |
| FTH_0808 | hypothetical protein | H |
| FTH_0950 | probable FAD/FMN-dehydrogenase | H |
| FTH_0959 | conserved hypothetical protein | H |
| FTH_0962 | probable ABC superfamily ATP binding cassette transporter, ABC protein | H |
| FTH_0983 | hypothetical protein | H |
| FTH_0994 | conserved hypothetical protein | H |
| FTH_1033 | hypothetical protein | H |
| FTH_1061 | hypothetical protein | H |
| FTH_1076 | hypothetical protein | H |
| FTH_1101 | hypothetical protein | H |
| FTH_1102 | hypothetical protein | H |
| FTH_1147 | hypothetical protein | H |
| FTH_1154 | hypothetical protein | H |
| FTH_1170 | conserved hypothetical protein | H |
| FTH_1188 | hypothetical protein | H |
| FTH_1190 | conserved hypothetical protein | H |
| FTH_1191 | hypothetical protein | H |
| FTH_1201 | conserved hypothetical protein | H |
| FTH_1211 | hypothetical protein | H |
| FTH_1212 | conserved hypothetical protein | H |
| FTH_1220 | conserved hypothetical protein | H |
| FTH_1231 | conserved hypothetical protein | H |
| FTH_1262 | hypothetical protein | H |
| FTH_1263 | conserved hypothetical protein | H |
| FTH_1336 | hypothetical protein | H |
| FTH_1480 | probable bifunctional transcriptional regulator/sugar kinase | H |
| FTH_1574 | conserved hypothetical protein | H |
| FTH_1586 | conserved hypothetical protein | H |
| FTH_1629 | conserved hypothetical protein | H |
| FTH_1653 | hypothetical protein | H |
| FTH_1684 | hypothetical protein | H |
| FTH_1826 | hypothetical protein | H |
| FTH_1866 | conserved hypothetical protein | H |
| FTH_0955 | type I site-specific deoxyribonuclease | HM |
| FTM_0876 | type III restriction enzyme, res subunit | HM |
| FTM_1290 | conserved hypothetical protein | HM |
| FTH_0147 | hypothetical protein | HT |
| FTH_0164 | conserved hypothetical protein | HT |
| FTH_0200 | conserved hypothetical protein | HT |
| FTH_0304 | hypothetical protein | HT |
| FTH_0336 | probable GNAT family acetyltransferase | HT |
| FTH_0471 | hypothetical protein | HT |
| FTH_0655 | hypothetical protein | HT |
| FTH_0796 | conserved hypothetical protein | HT |
| FTH_0810 | hypothetical protein | HT |
| FTH_1010 | hypothetical protein | HT |
| FTH_1057 | conserved hypothetical protein | HT |
| FTH_1060 | hypothetical protein | HT |
| FTH_1189 | conserved hypothetical protein | HT |
| FTH_1501 | conserved hypothetical protein | HT |
| FTH_1507 | hypothetical protein | HT |
| FTH_1621 | hypothetical protein | HT |
| FTH_1648 | hypothetical protein | HT |
| FTH_1713 | hypothetical protein | HT |
| FTH_1714 | probable LysR family transcriptional regulator | HT |
| FTH_1845 | conserved hypothetical protein | HT |
| FTT_0227c | hypothetical protein | HT |
| FTT_0254c | hypothetical protein | HT |
| FTT_0255c | hypothetical protein | HT |
| FTT_0401 | hypothetical protein | HT |
| FTT_0423 | hypothetical protein | HT |
| FTT_0424 | hypothetical protein | HT |
| FTT_0487 | hypothetical membrane protein | HT |
| FTT_0499 | conserved hypothetical protein | HT |
| FTT_0605c | hypothetical protein | HT |
| FTT_0612 | hypothetical protein | HT |
| FTT_0742 | hypothetical lipoprotein | HT |
| FTT_0888c | Type IV pili fiber building block protein | HT |
| FTT_1172c | cold shock protein (DNA-binding) | HT |
| FTT_1177c | hypothetical protein | HT |
| FTT_1480c | hypothetical protein | HT |
| FTT_1492c | hypothetical protein | HT |
| FTT_1597 | hypothetical protein | HT |
| FTT_1646 | D-tyrosyl-tRNA(Tyr) deacylase | HT |
| FTM_0509 | conserved hypothetical protein | HTM |
| FTM_0677 | IS1 transposase | HTM |
| FTT_1685 | hypothetical membrane protein | HTM |
| FTM_0529 | conserved hypothetical protein | M |
| FTM_0654 | fusion protein of transketolase 1, tktA, and Glyceraldehyde-3-phosphate dehydrogenase, gapA | M |
| FTM_0764 | hypothetical protein | M |
| FTM_0875 | type III restriction system endonuclease | M |
| FTM_0878 | conserved hypothetical protein | M |
| FTM_0934 | lipolytic enzyme, GDSL family | M |
| FTM_1222 | conserved hypothetical protein | M |
| FTM_1289 | conserved hypothetical protein | M |
| FTM_1557 | protein of unknown function with TPR repeat region and von Willebrand factor type A domain | M |
| FTM_1584 | hypothetical membrane protein | M |
| FTM_1735 | conserved hypothetical protein | M |
| FTT_0158c | hypothetical protein | T |
| FTT_0159c | hypothetical membrane protein | T |
| FTT_0199 | hypothetical protein | T |
| FTT_0200 | hypothetical protein | T |
| FTT_0267 | hypothetical protein | T |
| FTT_0274 | hypothetical membrane protein | T |
| FTT_0382 | hypothetical protein | T |
| FTT_0433 | hypothetical protein | T |
| FTT_0465 | hypothetical protein | T |
| FTT_0496 | conserved hypothetical protein | T |
| FTT_0500 | hypothetical protein | T |
| FTT_0520 | hypothetical protein | T |
| FTT_0522 | conserved hypothetical protein | T |
| FTT_0526 | hypothetical protein | T |
| FTT_0528 | hypothetical protein | T |
| FTT_0547 | hypothetical protein | T |
| FTT_0584 | hypothetical protein | T |
| FTT_0604 | conserved hypothetical membrane protein | T |
| FTT_0662c | conserved hypothetical protein | T |
| FTT_0677c | conserved hypothetical protein | T |
| FTT_0727 | conserved hypothetical protein | T |
| FTT_0741c | hypothetical protein | T |
| FTT_0744c | hypothetical protein | T |
| FTT_0752c | hypothetical protein | T |
| FTT_0754c | hypothetical membrane protein | T |
| FTT_0814c | hypothetical protein | T |
| FTT_0815c | hypothetical protein | T |
| FTT_0816c | chitin binding protein | T |
| FTT_0887c | hypothetical protein | T |
| FTT_0982 | hypothetical membrane protein | T |
| FTT_0983 | hypothetical membrane protein | T |
| FTT_0987 | hypothetical protein | T |
| FTT_0988 | hypothetical protein | T |
| FTT_1064 | hypothetical protein | T |
| FTT_1068c | hypothetical protein | T |
| FTT_1080c | hypothetical membrane protein | T |
| FTT_1122c | hypothetical lipoprotein | T |
| FTT_1211c | hypothetical protein | T |
| FTT_1491c | hypothetical protein | T |
| FTT_1541c | hypothetical protein | T |
| FTT_1595 | hypothetical protein | T |
| FTT_1623c | hypothetical protein | T |
| FTT_1657c | hypothetical protein | T |
| FTT_1784c | hypothetical protein | T |

**Supportive figure 1. Circular Genome view of *Francisella noatunensis subsp.orientalis.***

*
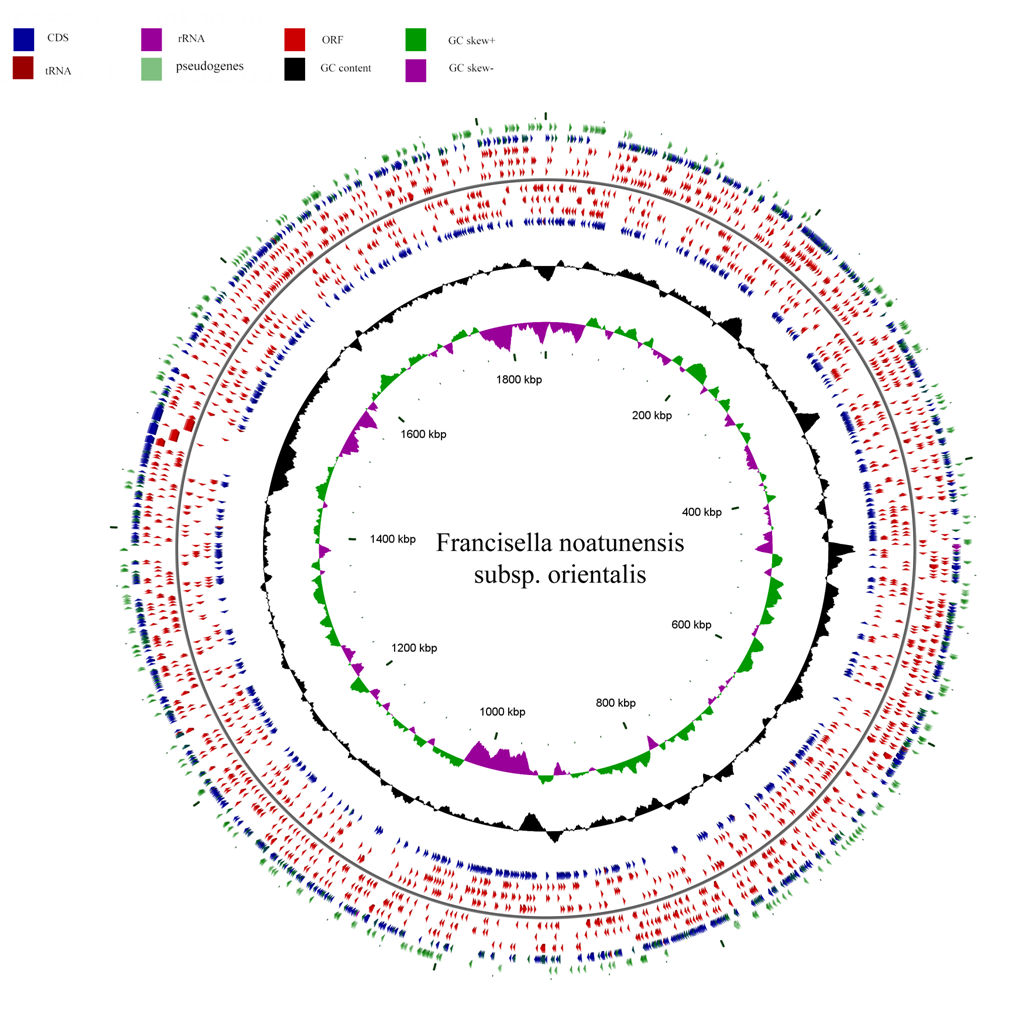
*

**Supportive figure 2. Map showing links between contigs.**


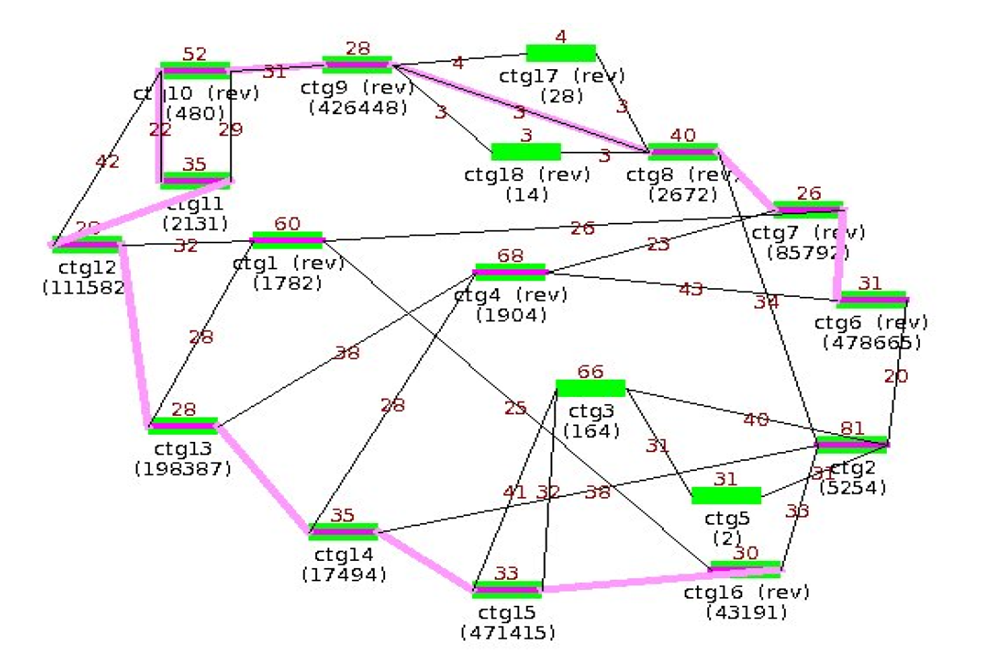


**Supportive Figure 3. Result from DAVID server showing over representation of functional terms in metabolic competent species.**

**
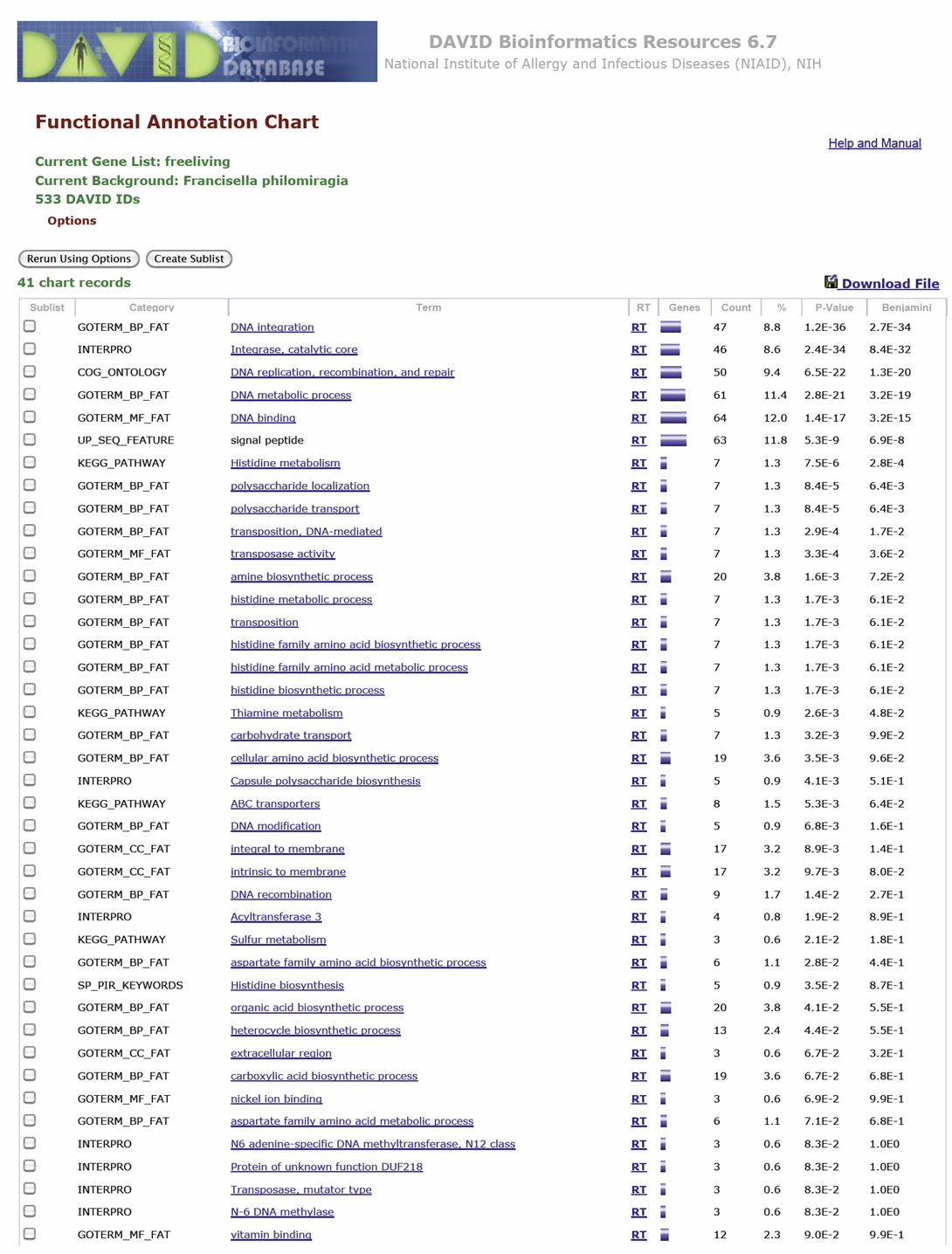
**

**Supportive Figure 4.**The given figure is one part of the comparison of *Francisella noatunensis subsp.orientalis* and *F.philomiragia subsp. philomiragia ATCC 25017* metabolic pathway. Red color indicates the reaction is shared between *F. noatunensis subsp. orientalis* and *F.philomiragia subsp. philomiragia*. Thin green lines indicates pathway hole in *F.philomiragia subsp. philomiragia.* Thick green line is the presence of reaction in *F.philomiragia subsp.philomiragia.*

**
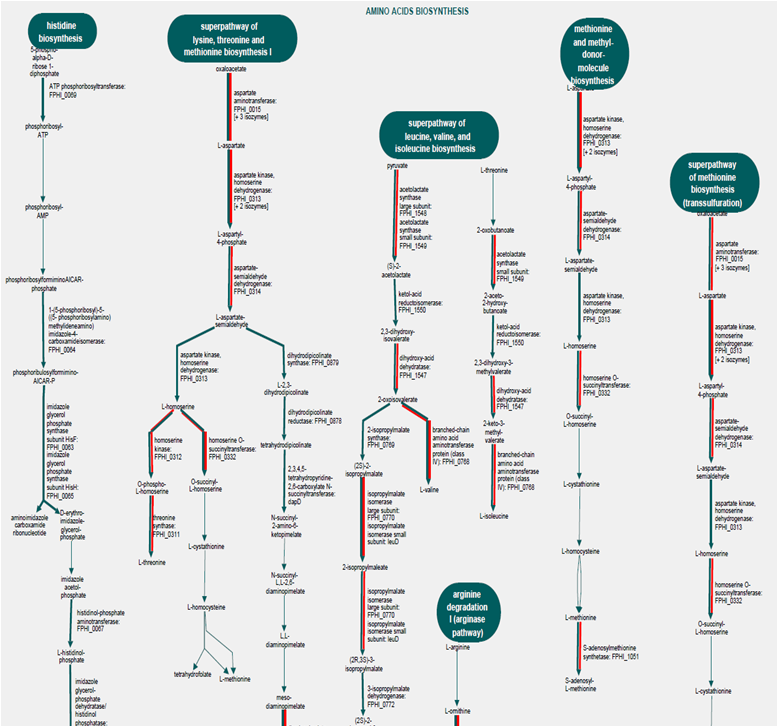
**
